# funRNA: a fungi-centered genomics platform for genes encoding key components of RNAi

**DOI:** 10.1186/1471-2164-15-S9-S14

**Published:** 2014-12-08

**Authors:** Jaeyoung Choi, Ki-Tae Kim, Jongbum Jeon, Jiayao Wu, Hyeunjeong Song, Fred O Asiegbu, Yong-Hwan Lee

**Affiliations:** 1 Center for Fungal Pathogenesis, Seoul National University, Seoul 151-921, Korea; 2 Fungal Bioinformatics Laboratory, Department of Agricultural Biotechnology, Seoul National University, Seoul 151-921, Korea; 3 Department of Forest Sciences, University of Helsinki, 00014 Helsinki, Finland; 4 Center for Fungal Genetic Resources, Plant Genomics and Breeding Institute, Research Institute for Agriculture and Life Sciences, Seoul National University, Seoul 151-921, Korea

## Abstract

**Background:**

RNA interference (RNAi) is involved in genome defense as well as diverse cellular, developmental, and physiological processes. Key components of RNAi are Argonaute, Dicer, and RNA-dependent RNA polymerase (RdRP), which have been functionally characterized mainly in model organisms. The key components are believed to exist throughout eukaryotes; however, there is no systematic platform for archiving and dissecting these important gene families. In addition, few fungi have been studied to date, limiting our understanding of RNAi in fungi. Here we present funRNA http://funrna.riceblast.snu.ac.kr/, a fungal kingdom-wide comparative genomics platform for putative genes encoding Argonaute, Dicer, and RdRP.

**Description:**

To identify and archive genes encoding the abovementioned key components, protein domain profiles were determined from reference sequences obtained from UniProtKB/SwissProt. The domain profiles were searched using fungal, metazoan, and plant genomes, as well as bacterial and archaeal genomes. 1,163, 442, and 678 genes encoding Argonaute, Dicer, and RdRP, respectively, were predicted. Based on the identification results, active site variation of Argonaute, diversification of Dicer, and sequence analysis of RdRP were discussed in a fungus-oriented manner. funRNA provides results from diverse bioinformatics programs and job submission forms for BLAST, BLASTMatrix, and ClustalW. Furthermore, sequence collections created in funRNA are synced with several gene family analysis portals and databases, offering further analysis opportunities.

**Conclusions:**

funRNA provides identification results from a broad taxonomic range and diverse analysis functions, and could be used in diverse comparative and evolutionary studies. It could serve as a versatile genomics workbench for key components of RNAi.

## Background

RNA interference (RNAi), a term first coined in research on *Caenorhabditis elegans*, was originally thought to be a host defense mechanism against invasion of viral genomes or transposable elements [1]. However, several molecular studies revealed that it is also involved in diverse cellular, developmental, and physiological processes [2-5]. Gene silencing by RNAi begins with recognition of aberrant RNA (aRNA) or introduction of double-stranded RNA (dsRNA), such as viral genomes. RNA-dependent RNA polymerase (RdRP) is responsible for the generation of dsRNA from aRNA. Dicer slices dsRNA into small (21-25 nt) pieces. Argonaute then acts on these fragments by forming an RNA-induced silencing complex (RISC), which is subsequently guided to target mRNAs, resulting in gene silencing.

In fungi, molecular characterization of genes encoding RNAi components has been intensively studied in *Neurospora crassa *and *Schizosaccharomyces pombe*. In *N. crassa*, there are two characterized post-transcriptional gene silencing (PTGS) mechanisms: quelling in a vegetative state and meiotic silencing by unpaired DNA (MSUD) at the sexual stage. Inspired by transcriptional down-regulation in albino-1 (*al-1*) or albino-3 (*al-3*) gene-overexpressing strains of *N. crassa *[6-8], quelling-defective (*qde*) mutants including *qde-1*, *qde-2*, and *qde-3*, as well as the Dicer-encoding genes *dcl-1 *and *dcl-2*, were characterized [9-12]. Differences between the two pathways include the occurrence of MSUD during prophase in meiosis I and the proteins involved in the pathways. In quelling, RdRP QDE-1 and Argonaute QDE-2 are required, whereas MSUD utilizes their paralogs, SAD-1 (suppressor of ascus dominance 1) and SMS-2 (suppressor of meiotic silencing 2), respectively [13,14]. In filamentous fungi, such as the abovementioned *N. crassa*, gene silencing mediated by RNAi occurs post-transcriptionally. On the other hand, in *S. pombe*, RNAi contributes to transcriptional gene silencing through heterochromatin formation [15].

Despite the importance of this universal machinery in eukaryotes, many studies on RNAi focused only on functional, physiological, and molecular aspects, rather than comparative genomics. It is known that particular fungal taxa, for example budding yeasts, do not have the key components of RNAi [16]. Hence a systematic, extensive identification and evolution analysis are needed to determine the clear distribution of the genes and to trace their evolutionary histories. Furthermore, considering that Argonaute-encoding genes were found in a few non-eukaryotic species [17], the taxonomic distribution and phyletic trajectory of these important genes could tell us more about their ancestral origin. As a solution, we developed funRNA http://funrna.riceblast.snu.ac.kr/ to provide a gene catalogue based on 1,440 genomes and a comparative, evolutionary genomics platform for the genes encoding Argonaute, Dicer, and RdRP.

In this paper, we discuss the following: i) the taxonomic distribution of the key components of RNAi; ii) sequence analysis of predicted RdRPs by multiple sequence alignment; iii) auxiliary domain variation in Dicers; iv) evolutionary analysis of the putative genes encoding Argonautes by gene duplication and loss; and v) database and web functionalities available on the funRNA website.

## Results and discussion

### Content and distribution of the identified genes encoding Argonaute, Dicer, and RdRP

**Figure 1:**
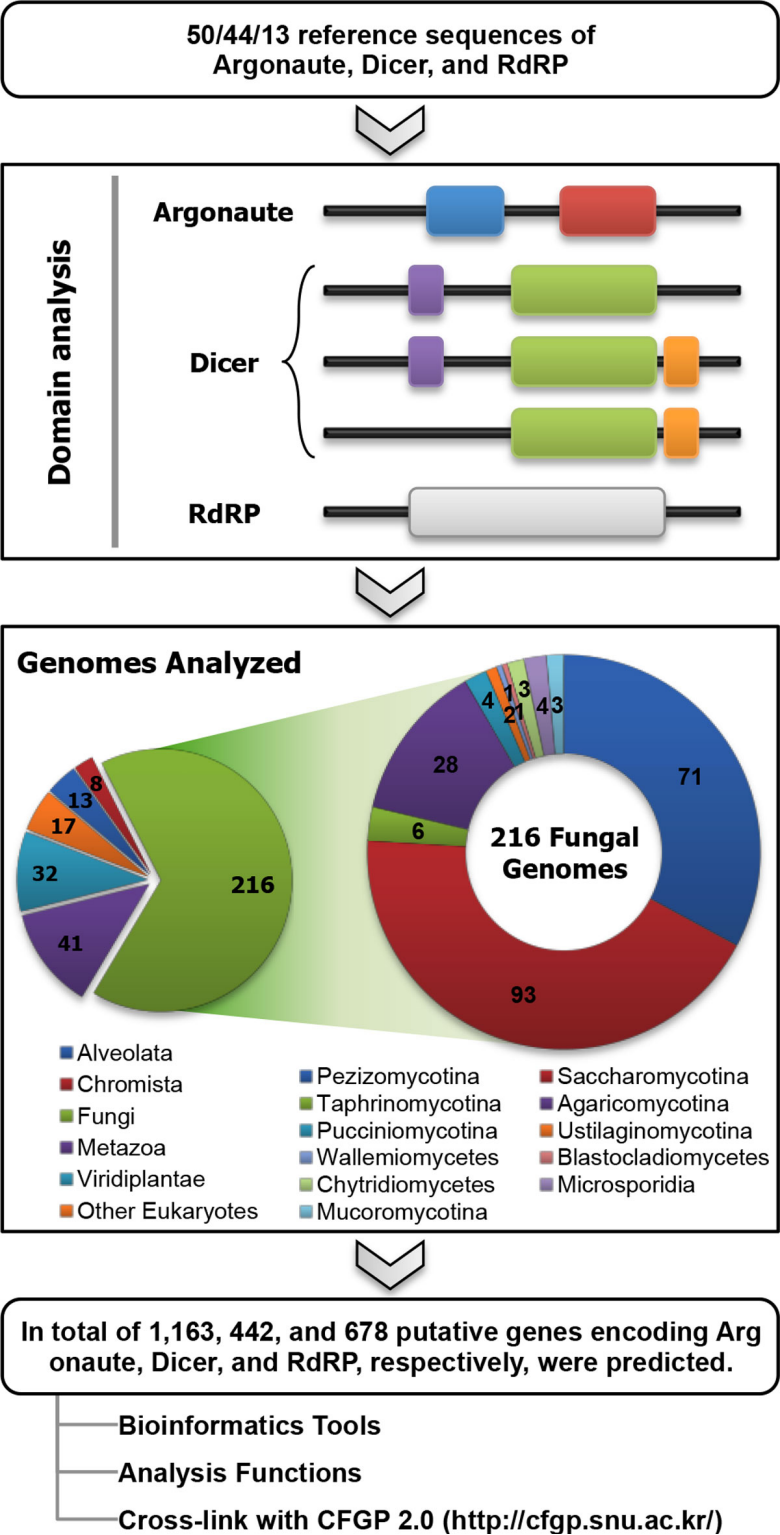
**Identification pipeline for funRNA**. The identification pipeline for funRNA consists of two steps: i) defining domain profiles from protein sequences encoded by the reference sequences; and ii) scanning 1,440 proteomes with domain profiles for Argonaute, Dicer, and RdRP. In "Domain analysis", colored boxes indicate essential domains: blue, IPR003100 (Argonaute/Dicer protein, PAZ); red, IPR003165 (Stem cell self-renewal protein Piwi); purple, IPR005034 (Dicer double-stranded RNA-binding fold); green, IPR000999 (Ribonuclease III); orange, IPR001159 (Double-stranded RNA-binding); and gray, IPR007855 (RNA-dependent RNA polymerase, eukaryotic type). In addition, sequences collected from funRNA can be subjected to bioinformatics analysis on the funRNA website as well as in CFGP 2.0 by data exchange through the Favorite Browser.

In order to predict putative genes, 1,440 genomes were searched using protein domain profiles (Figure 1; see Methods for details). 1,163 Argonaute-encoding genes, 442 Dicer-encoding genes, and 678 RdRP-encoding genes were predicted (Additional file 1). In order to evaluate the accuracy of the pipeline, a test set was prepared by retrieving sequences annotated as Argonaute, Dicer, and RdRP from UniProtKB/TrEMBL [18]. Assuming that the annotation provided by UniProtKB/TrEMBL is correct, the funRNA pipeline correctly captured 93.50% of the test set. This result supports the accuracy and robustness of the funRNA gene identification pipeline. According to the prediction results, the average numbers of genes encoding Argonaute and Dicer were significantly higher in Metazoa and Viridiplantae than in Fungi (t-test: *P *≤ 2.53e^-6 ^for Argonaute and *P *≤ 7.38e^-5 ^for Dicer). In the case of RdRP, the Viridiplantae kingdom presented the largest average number of genes (5.09), followed by Fungi (2.04) and Metazoa (1.27) (t-test: *P *≤ 2.45e^-4^). No genes encoding Argonaute, Dicer, or RdRP were detected in 1,059 and 51 proteomes of bacteria and archaea, respectively. However, two archaeal species (*Methanocaldococcus jannaschii *DSM 2661 and *Pyrococcus furiosus *DSM 3638) and one bacterial species (*Aquifex aeolicus *VF5) were predicted to have an Argonaute-encoding gene. In fact, PfAgo, an Argonaute found in *P. furiosus *DSM 3638, has been structurally characterized by X-ray crystallography [17], and was correctly captured by the pipeline.

**Figure 2:**
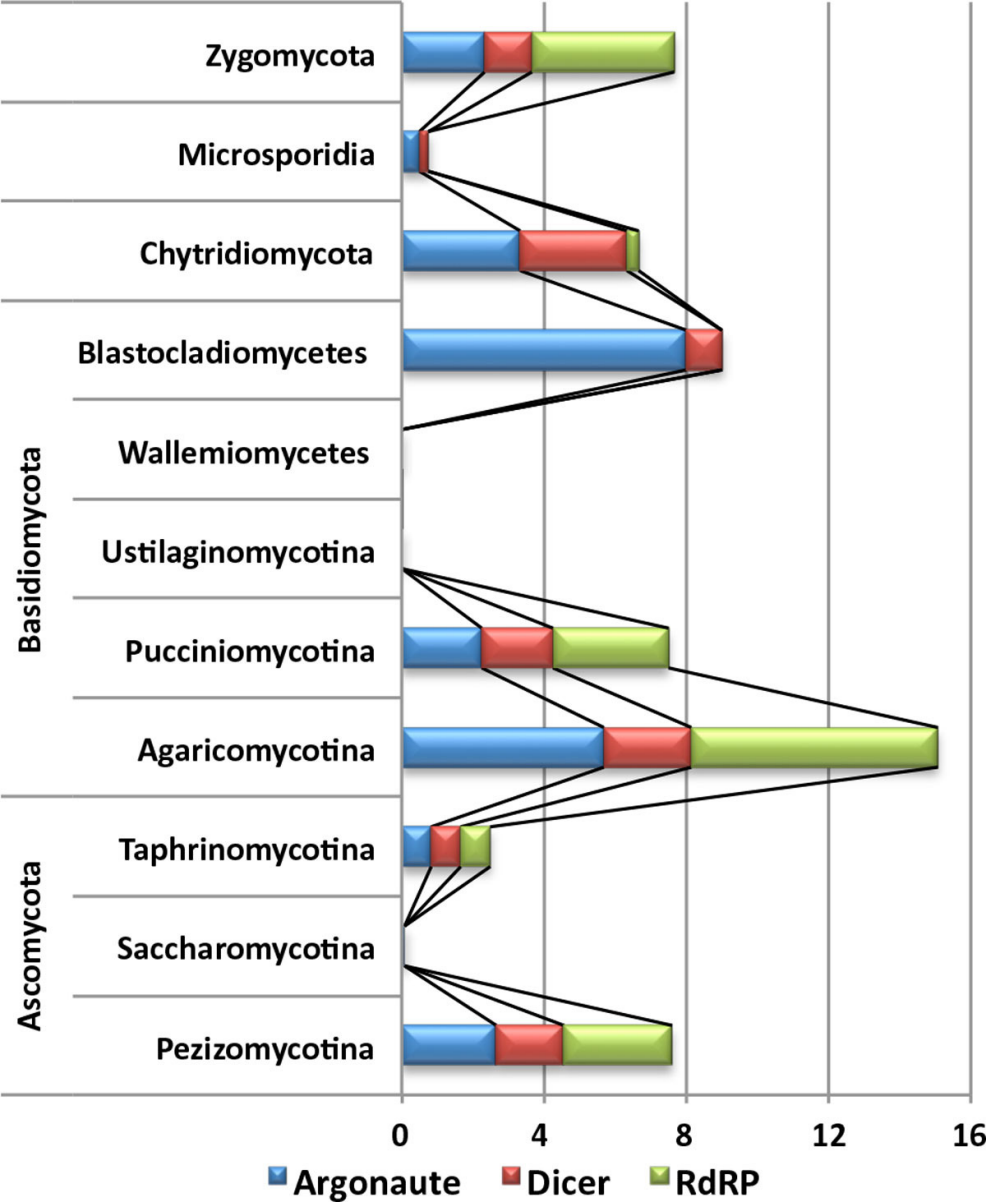
**Distribution of the average number of genes across the taxonomic spectrum**. The average numbers of gene families for each fungal taxon is shown as a cumulative bar chart. The sizes of the blue, red, and green areas in a stack indicate the average number of putative genes encoding Argonaute, Dicer, and RdRP, respectively.

In fungi, species belonging to the subphylum Agaricomycotina showed a higher number of genes in all gene families than any other fungal subphylum, with 5.68 Argonaute, 2.46 Dicer, and 6.93 RdRP genes on average (Figure 2 andTable 1). Putative genes were not predicted in the species belonging to the subphylum Ustilaginomycotina, in agreement with previous reports [19,20]. In the phylum Ascomycota, the majority of genes were found in species belonging to the subphylum Pezizomycotina. Species belonging to the subphylum Saccharomycotina, including five genomes of *Candida *spp., had only a few genes encoding Argonaute and no genes for Dicer and RdRP. Although a recent paper reported the presence of RNAi in *Saccharomyces castellii *and *C. albicans*, these species use non-canonical Dicers to generate small interfering RNAs [21]. Meanwhile, *Schizosaccharomyces *spp., belong to the subphylum Taphrinomycotina, had one gene for each of three gene families (Additional file 1).

**Table 1:** Summary of the average number of genes per genome across the taxonomic spectrum.

Kingdom	Phylum	Subphylum	Argonaute^*^	Dicer^*^	RdRP^*^
Chromalveolata			0	0	0.15 (0-2)

Chromista			2.75 (0-6)	0.25 (0-1)	0.88 (0-3)

Fungi	Ascomycota	Pezizomycotina	2.66 (1-7)	1.9 (0-3)	3.04 (2-5)
		Saccharomycotina	0.08 (0-1)	0	0
		Taphrinomycotina	0.83 (0-1)	0.83 (0-1)	0.83 (0-1)
	Basidiomycota	Agaricomycotina	5.68 (0-10)	2.46 (0-4)	6.93 (0-14)
		Pucciniomycotina	2.25 (2-3)	2 (0-5)	3.25 (1-5)
		Ustilaginomycotina	0	0	0
		Wallemiomycetes^**^	0	0	0
		Blastocladiomycetes^**^	8	1	0
	Chytridiomycota	N/D	3.33 (2-6)	3.00 (3)	0.33 (0-1)
	Microsporidia	N/D	0.50 (0-1)	0.25 (0-1)	0
	Zygomycota	Mucoromycotina	2.33 (2-3)	1.33 (1-2)	4.00 (3-5)

Metazoa			9.88 (1-49)	2.24 (0-7)	1.27 (0-9)

Viridiplantae	Chlorophyta		0.90 (0-2)	0.40 (0-2)	0.30 (0-1)
	Streptophyta		13.55 (6-25)	4.86 (0-11)	7.27 (3-14)

Other eukaryotes			1.76 (0-14)	0.29 (0-3)	0.76 (0-4)

^* ^Range of number of genes is shown in parentheses.

^** ^Class name is shown if no subphylum name is assigned.

In *Plasmodium *spp., no genes were predicted to encode the key players of RNAi. This is in accordance with previous studies performed with protozoan parasites including *Trypanosoma cruzi*, *Leishmania major*, *L. donovani*, and *Plasmodium *spp. [22,23]. The only exception is the RNA-mediated gene silencing in *P. falciparum *[24,25]. Accordingly, it was speculated that *Plasmodium *spp. might possess a non-canonical RNAi pathway that is yet to be characterized [26].

While the three genes were frequently predicted in two fungal subphyla, Pezizomycotina and Agaricomycotina, the number of genes in the latter subphylum varied more than that in the former. The greatest variance was found in the genes encoding RdRP. The number of RdRP genes in the Pezizomycotina species ranged from two to five, with a standard deviation of 0.64; for Agaricomycotina the range was zero to 14, with a standard deviation of 3.63. Interestingly, no putative genes encoding RdRP were found in two metazoan phyla, Arthropoda (except for in *Ixodes scapularis*, the blacklegged tick) and Chordata, to which fruit flies and humans belong, respectively. Considering the possibility that virus-encoded RdRPs may play a role in RNAi-like antiviral activity in plants [27], we speculated that the same could be happening in *Drosophila *spp. and mammals. This was supported by the fact that mouse oocytes with a horizontally transferred RdRP from a virus exhibited RNAi [28]. By contrast, worm species belonging to the phylum Nematoda had multiple genes with copy numbers ranging from three to eight. Meanwhile, in the Viridiplantae kingdom, a clear distinction in the number of genes was found between the Chlorophyta and Streptophyta phyla. Streptophyta species had higher average numbers of the three genes (13.55 for Argonaute, 4.86 for Dicer, and 7.27 for RdRP) than Chlorophyta (0.90, 0.40, and 0.30, respectively) (t-test: *P *= 1.60e^-10^, 2.13e^-08^, and 1.69e^-10^, respectively). In *Chlamydomonas reinhardtii*, which belongs to the Chlorophyta, it was presumed that the absence of an RdRP gene is a reflection of its minimalistic genomic nature, and that it thus only exhibits the essential RNAi phenomenon [29]. It was also speculated that *C. reinhardtii *only recognizes dsRNA to trigger RNAi, since the transformation of single-stranded RNA favors non-homologous recombination [30]. Notably, *Lotus japonicus *was predicted to have no genes encoding Dicer. In fact, two genes encoding Dicer-like proteins were predicted to have RNase III and dsRNA-binding domains. They had only one RNase III domain, while canonical Dicers are known to contain two separate RNase III domains. Because experimental evidence shows that functional RNAi is present in *L. japonicus *[31,32], the two genes may encode real Dicers with a simpler domain structure. In addition, monocot plants tend to have more Argonaute genes than dicots. Actually, orthologs of Dicer-like genes in *Oryza sativa *were found in other monocot plants, such as *Zea mays *and *Saccharum officinarum*, but not in *Arabidopsis thaliana*, supporting gene duplication after monocot and dicot divergence [33].

### Evolutionary history of gene duplication/loss and active sites residues in Argonautes

The identification results showed that Argonaute-encoding genes were found in many fungal species in the subphyla Pezizomycotina and Agaricomycotina, as well as in plants and animals (Additional file 1). In order to elucidate evolutionary footprints, reconciliation analysis was performed with a species tree and an Argonaute gene tree. A total of 34 species predicted to have Argonaute-encoding genes were subjected to the analysis, which covered species belonging to multiple kingdoms, including Fungi, Viridiplantae, Metazoa, Bacteria, and Archaea (Table 2). Massive gene duplication events were found in the animal and plant species. In fungi, however, duplications were found only in basidiomycetes and two ascomycetes.

**Table 2:** List of species selected for sequence analysis of RdRPs and reconciliation analysis of Argonautes.

Species name	Taxonomy	Argonaute gene	RdRP gene
*Methanocaldococcus jannaschii *DSM 2661	Archaea>Euryarchaeota>N/D	1	0
*Pyrococcus furiosus *DSM 3638	Archaea>Crenarchaeota>N/D	1	0
*Aquifex aeolicus *VF5	Bacteria>Aquificae>N/D	1	0
*Aspergillus fumigatus *Af293	Fungi>Ascomycota>Pezizomycotina	2	2
*Aspergillus nidulans*	Fungi>Ascomycota>Pezizomycotina	1	2
*Botrytis cinerea*	Fungi>Ascomycota>Pezizomycotina	2	2
*Coccidioides immitis *RS	Fungi>Ascomycota>Pezizomycotina	3	3
*Colletotrichum graminicola *M1.001	Fungi>Ascomycota>Pezizomycotina	2	3
*Fusarium graminearum*	Fungi>Ascomycota>Pezizomycotina	2	5
*Fusarium oxysporum*	Fungi>Ascomycota>Pezizomycotina	5	5
*Histoplasma capsulatum *H88	Fungi>Ascomycota>Pezizomycotina	2	3
*Magnaporthe oryzae *70-15	Fungi>Ascomycota>Pezizomycotina	3	3
*Mycosphaerella graminicola*	Fungi>Ascomycota>Pezizomycotina	4	2
*Neurospora crassa*	Fungi>Ascomycota>Pezizomycotina	2	3
*Podospora anserina*	Fungi>Ascomycota>Pezizomycotina	2	4
*Candida albicans*	Fungi>Ascomycota>Saccharomycotina	1	0
*Schizosaccharomyces pombe*	Fungi>Ascomycota>Taphrinomycotina	1	1
*Heterobasidion irregulare *TC 32-1	Fungi>Basidiomycota>Agaricomycotina	7	7
*Laccaria bicolor*	Fungi>Basidiomycota>Agaricomycotina	6	6
*Phanerochaete chrysosporium*	Fungi>Basidiomycota>Agaricomycotina	6	8
*Serpula lacrymans*	Fungi>Basidiomycota>Agaricomycotina	6	6
*Cryptococcus neoformans *var. *grubii *H99	Fungi>Basidiomycota>Agaricomycotina	1	1
*Melampsora laricis-populina*	Fungi>Basidiomycota>Pucciniomycotina	2	5
*Puccinia graminis*	Fungi>Basidiomycota>Pucciniomycotina	2	5
*Allomyces macrogynus*	Fungi>Blastocladiomycota>N/D	8	0
*Batrachochytrium dendrobatidis *JAM81	Fungi>Chytridiomycota>N/D	2	0
*Phycomyces blakesleeanus*	Fungi>Zygomycota>Mucoromycotina	2	4
*Rhizopus oryzae*	Fungi>Zygomycota>Mucoromycotina	2	5
*Phytophthora infestans*	Chromista>Oomycota>Oomycotina	5	1
*Arabidopsis thaliana*	Viridiplantae>Streptophyta>N/D	14	6
*Oryza sativa*	Viridiplantae>Streptophyta>N/D	25	6
*Drosophila melanogaster*	Metazoa>Arthropoda>N/D	12	0
*Caenorhabditis elegans*	Metazoa>Nematoda>N/D	31	4
*Homo sapiens*	Metazoa>Chordata>Craniata	17	0

**Figure 3:**
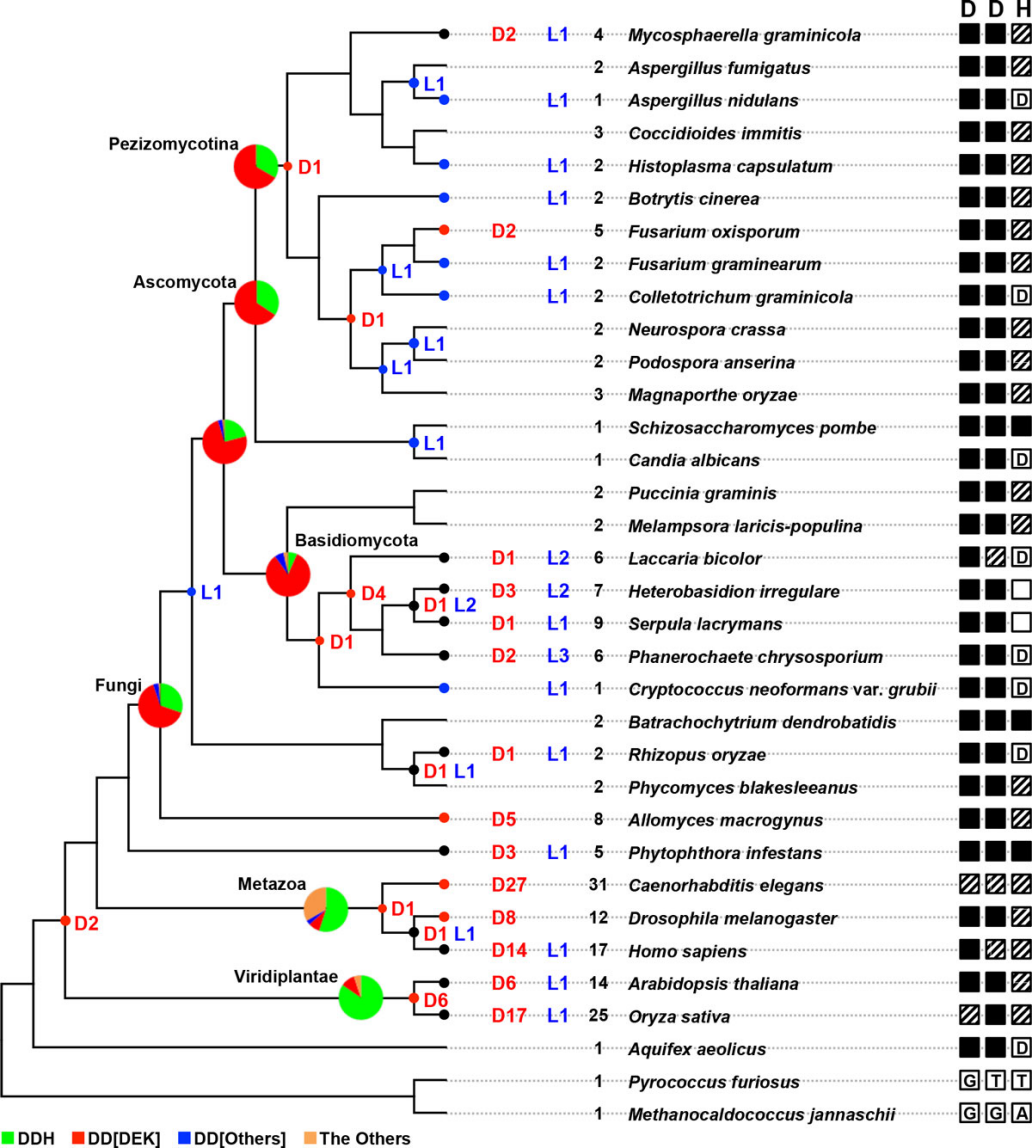
**Duplication and loss of Argonaute genes and variation of the catalytic motif**. Gene duplication and loss events were estimated by reconciliation analysis. Red and blues dots are shown at internal nodes if duplication and loss were predicted, respectively. Black dots indicate nodes where both duplication and loss were discovered. Numbers of species-level duplication and loss events, and the number of putative genes encoding Argonaute, are shown between the tree and species name. In the rightmost column, amino acid variation of the DDH motif is shown with symbols: i) filled squares indicate that all the genes in the corresponding species had the conserved reference residue; ii) shaded squares indicate existence of the conserved residue and variants; iii) empty squares indicate variants without the conserved residue; and iv) single-letter amino acid codes indicate conserved residues, but not the reference amino acid. For the complete list of partial alignments near each amino acid, see Additional file 2. Pie charts shown in the internal nodes display the distribution of DDH motif variants for each taxon specified. The proportion of genes containing the conserved DDH motif is shown in green; H substituted by D, E, or K is shown in red; H substituted by another amino acid is shown in blue; and other variants are shown in orange.

Catalytic amino acid residues for Argonaute slicer activity were characterized in previous studies [17,34,35]. Argonaute sequences found in *Homo sapiens*, *A. thaliana*, *D. melanogaster*, *S. pombe*, and *C. elegans *were analyzed for catalytic residues, or the DDH motif [36]. In addition, functional variants of the DDH motif were experimentally identified, giving a relaxed motif definition, DD[HDEK] [34,37]. Archaea, as the most divergent species evolutionarily, have a totally different composition of active site amino acids compared to other species (Figure 3). The DD[HDEK] motif was found in Argonaute sequences from a bacterium (*Aquifex aeolicus*), fungi, plants, and animals. In fungi, the first two residues were well conserved. The third residue was variable, but was predominantly aspartic acid (Figure 3 and Additional file 2). Dicers in *C. elegans*, *H. sapiens*, and *O. sativa *have been through more species-level duplications, which possibly resulted in greater residue variation in the catalytic motif. The DDH triad was the most frequent motif in Argonautes of animals and plants; most fungus Argonautes had a DD[DEK] motif. "The Others", sequences showing aligned residues other than DD[HDEK], were much more common in animal species, especially *C. elegans*, possibly due to gene diversification resulting from a number of species-level duplication events.

### Differential distribution of accessory domains in putative Dicers

In plants, the copy numbers of genes encoding Dicer-like proteins increased during their evolution, with diversification occurring by duplication and transposition [38]. In fact, our data also show diversification of Dicer-like genes in plants, including *Glycine max*, *Z. mays*, *O. sativa*, *Brachypodium distachyon*, and *A. thaliana*. Diversification of Dicer often presented as alternatively spliced genes that produce multiple products (Additional file 3). Interestingly, this was true for species belonging to the phylum Streptophyta, but not for Chlorophyta. Only 3 of 10 Chlorophyta species were predicted to have one or two Dicer-encoding genes, while the other seven were not predicted to have any Dicer genes (Additional file 1). This confirms the previous finding that diversification of Dicers in plants occurred before the divergence of monocot and dicot plants, but after the divergence of green algae and plants [38]. Even though a few fungal genomes provided alternatively spliced transcript information, fungal Dicers showed no evidence of diversification by alternative splicing. In *N. crassa*, *Magnaporthe oryzae *70-15, and *Cryptococcus neoformans *var. *grubii *H99, for example, no alternatively spliced form was found for the putative Dicer-encoding genes.

Besides the essential RNase III and dsRNA-binding domains (Table 3), the other 50 additional domains were not universally present in the Dicers in plants, animals, and fungi (Additional file 4). On average, each had 5.28 additional domains other than the essential ones. The genes of the Viridiplantae species showed the highest number of additional domains (6.41), followed by those of Metazoa (5.12) and Fungi (4.87). The top six most frequent additional domains were IPR001650 (Helicase, C-terminal), IPR014001 (DEAD-like helicase), IPR011545 (DNA/RNA helicase, DEAD/DEAH box type, N-terminal), IPR003100 (Argonaute/Dicer protein, PAZ), IPR006935 (UvrABC complex, subunit B), and IPR014720 (Double-stranded RNA-binding-like) (Additional file 4). Among the 50 domains, the distribution of three dsRNA-binding domains (InterPro accession numbers IPR001159, IPR005034, and IPR014720) varied across the taxonomic spectrum. All fungal Dicers had an IPR005034 domain, but those in *Dictyostelium *spp., animal, and plant species often lacked the domain (Additional file 4). The IPR001159 domain was present in 20.74% (28 of 135) of the predicted sequences from species belonging to the subphylum Pezizomycotina. By contrast, there was no Dicer containing the IPR001159 domain in species belonging to the subphylum Agaricomycotina. Meanwhile, Metazoa and Viridiplantae species had one or both of the IPR001159 and IPR005034 domains. The IPR014720 domain was only detected in genes from two fungi (*Trichoderma atroviride *and *Mucor circinelloides*), while 46.31% (94 of 203) of metazoan and plant Dicers were predicted to have the domain. Interestingly, the PAZ domain (IPR003100), named after the proteins Piwi, Argonaute, and Zwille, was rarely found in fungi (nine out of 232 proteomes), but more often in metazoan and plant species (51/92 and 99/111, respectively). Although the three-dimensional structure of the PAZ domain has been resolved [39-41], its function is not clear, although it has been speculated that it may mediate the formation of complexes between proteins of the Piwi and Dicer families by heterodimerization [42]. Future research may focus on the functionality of Dicers without the PAZ domain to demonstrate the essentiality of the domain in fungi.

**Table 3:** Domain profile definitions used in funRNA

Gene family	InterPro accession number	Domain description	Number of genes^*^	Number of genomes^*^
Argonaute	IPR003100	Argonaute/Dicer protein, PAZ	1,163 (396)	209 (122)
	IPR003165	Stem cell self-renewal protein Piwi		

Dicer	IPR000999	Ribonuclease III	442 (232)	180 (111)
	IPR001159^**^	Double-stranded RNA-binding		
	IPR005034^**^	Dicer double-stranded RNA-binding fold		

RdRP	IPR007855	RNA-dependent RNA polymerase, eukaryotic type	678 (441)	157 (111)

^* ^Numbers of fungal genes/genomes are shown in parentheses.

^** ^Genes containing one of two double-stranded RNA-binding domains were predicted to be Dicer-encoding genes.

Differences in domain composition were also reflected in a phylogenetic tree that was constructed using the 442 Dicer sequences (Additional file 3). It is noteworthy that the tree was taxonomically divided into four clades: two Metazoa-dominant clades, one plant-dominant clade, and one fungus-dominant clade. In plant species, isoform products were grouped together closely, supporting the diversification reported previously [38]. Interestingly, the putative Dicers from metazoan species formed two distinct clades, one containing minimal domains and the other containing multiple additional domains (Additional files 3 and 4). The two Metazoa-dominant clades suggest that minimal Dicer could be the ancestral form, which acquired additional domains during the evolution of individual organisms. Most of the fungal and plant Dicers possessed multiple additional domains.

### Structural conservation analysis of residues in catalytic regions of RdRPs in fungi

In *N. crassa*, a 2.3-Å-resolution crystal structure of an RdRP (QDE-1) was characterized [43]. QDE-1 was structurally aligned with the protein sequences of bacterial and yeast polymerases. Structurally conserved catalytic motifs, including double-psi β-barrels (DPBB1 and 2), with multiple invariant residues were found. To test the conservation of such amino acid residues, a multiple sequence alignment was performed with 84 putative RdRP sequences from selected fungal proteomes (Table 2), including QDE-1. When counting the residues with 70% or higher conservation, 13 and 22 residues were found to be conserved in DPBB1 and DPBB2 based on the positions in QDE-1, respectively. Some were also reported to be conserved in a previous study. For example, three aspartic acid residues (D) located at positions 1,007, 1,009, and 1,011 in QDE-1 were conserved in 84.52% of the sequences analyzed. The high conservation of these three aspartic acid residues reflects their importance in binding Mg^2+ ^as a cofactor. Double glycine (G at positions 1,005 and 1,006 in QDE-1) was found in 83.33% of analyzed sequences, although not in bacterial and yeast polymerases [43] (Additional file 5).

## Utility

### Web utility

**Figure 4:**
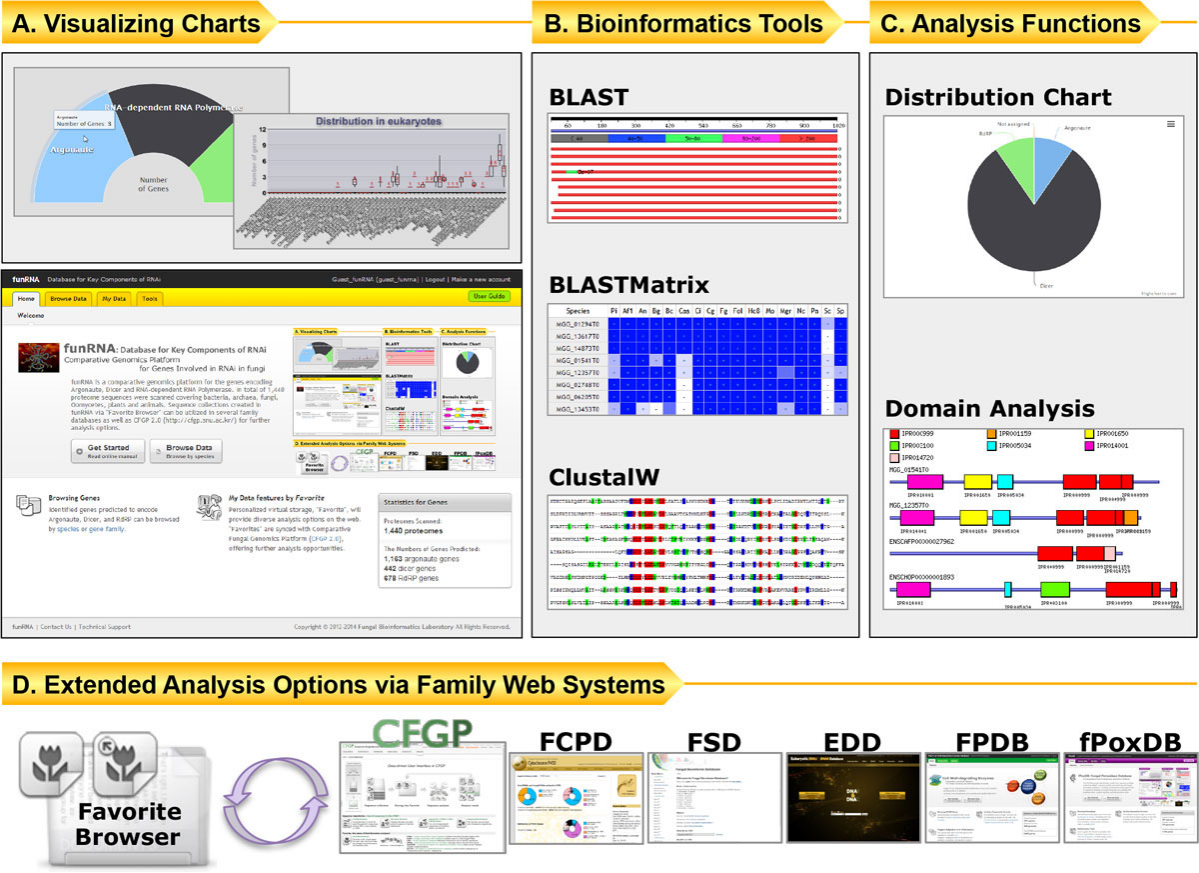
**Functionalities of the funRNA website**. A) Web interface of funRNA displays graphical charts for better recognition of the distribution of genes. B) Tools including similarity search tools (BLAST and BLASTMatrix) and a multiple sequence alignment tool (ClustalW) are provided via the Favorite Browser. C) Protein domain analysis can be conducted with the sequences collected in Favorites. D) Users' sequence collections can be further analyzed by the tools available in CFGP 2.0 and other sister databases.

To provide a user-friendly interface, funRNA adopted the Data-driven User Interface powered by the Comparative Fungal Genomics Platform 2.0 (CFGP 2.0; http://cfgp.snu.ac.kr/) [44]. The genes identified by the pipeline can be browsed by species or gene family. The reference sequences used in pipeline construction are also available. The detail information page for each gene shows the gene structure, sequence information, domain structure, GO terms, information on similarity to the reference sequences, and results from seven additional bioinformatics programs. The statistics page of "Species Browser" provides a kingdom-/subphylum-level summary, giving a glimpse into the macro-taxonomic distribution. funRNA also provides analysis functions, including: i) sequence similarity searches (BLAST [45] and BLASTMatrix [44]); ii) multiple sequence alignment (ClustalW [46]) with full-length or domain regions; and iii) protein domain analysis and download functions (Figure 4).

### Extended analyses through sister web-based systems

funRNA supports "Favorite Browser", a personalized virtual storage and analysis hub that was originally developed in CFGP 2.0 [44]. Sequences archived in funRNA have the same identifiers as those used in CFGP 2.0, enabling flexible data exchange with CFGP 2.0, as well as with sister databases [47-50]. In Favorite Browser in CFGP 2.0, 27 bioinformatics tools are currently available, providing broader analysis options for the sequences collected in funRNA. For example, a sequence collection created in funRNA could be analyzed in Favorite Browser in CFGP 2.0 to find conserved motifs by using the MEME program [51].

## Conclusions

funRNA is a web-based workbench that provides an analysis environment for the key components of RNAi. funRNA provides: i) a putative gene archive from 1,440 proteomes over a wide taxonomic range; ii) graphs and summary tables for an overview of the target gene families; iii) detailed information about the predicted genes; iv) job submission forms for bioinformatics tools; and v) Favorite synchronization with CFGP 2.0 and sister databases to offer further analysis. In addition, diverse comparative analyses can be conducted, such as the analyses shown in this paper. In summary, funRNA is a useful resource for comparative and evolutionary genomics analyses of Argonaute, Dicer, and RdRP genes.

## Methods

### Establishment of protein domain profiles

In order to determine protein domain profiles for the genes encoding Argonaute, Dicer, and RdRP, annotated protein sequences were retrieved from the UniProtKB/SwissProt database [18]. In total, 50, 44, and 13 sequences belonging to the respective gene families were subjected to domain profiling using InterPro scan (version 4.8) [52]. For each gene family, commonly shared domains were determined and used for prediction of putative genes (Table 3). The domain profiles acquired from the reference sequences were consistent with previous findings [53,54]. According to previous studies, Argonautes contain PIWI and PAZ domains [36], and fungal Dicer and Dicer-like proteins have RNase III and double-stranded RNA binding domains [53]. For Dicer identification, sequences with only one RNase III domain were discarded from the final prediction to improve the results. All sequences used to construct the pipeline are available from "Reference Sequences" under the "Browse Data" menu.

### Preparation of proteome sequences to be searched

To identify genes involved in small RNA processing, 1,440 proteomes were scanned with the protein domain profiles (Additional file 1). The target proteomes included 221 fungal/Oomycete genomes, as well as 1,060 bacterial, 53 archaeal, 32 plant, and 41 metazoan proteomes to investigate evolutionary traces in other kingdoms (Additional file 1). All the proteome sequences were obtained from the standardized genome data warehouse in CFGP 2.0 http://cfgp.snu.ac.kr/[44].

### Evaluation of the pipeline

To evaluate the gene identification pipeline, sequences annotated as Argonaute, Dicer, and RdRP were obtained from UniProtKB/TrEMBL [18]. They did not include sequences used in domain profile determination. The test set consisted of 425 Argonaute, 209 Dicer, and 227 RdRP protein sequences. The sequences were scanned using InterPro scan [52], and searched with the funRNA domain profiles to assess the accuracy. Considering the average length of the sequences used in defining the domain profiles (942 aa for Argonaute, 1,575 aa for Dicer, and 1,081 aa for RdRP), sequences shorter than 500 aa were discarded from the test set.

### Assessment of gene duplication and loss

A species phylogeny was created by using CVTree (version 4.2.1; source code distribution) [55]. Whole proteome sequences of the target species were used as the input, and K-tuple length was set to seven, which is known to be optimal for fungal phylogeny construction [56,57]. The output distance matrix was converted into a neighbor-joining tree by using *neighbor *in the PHYLIP package (version 3.69) [58]. To build gene trees, multiple sequence alignment was performed by using MUSCLE in MEGA6 [59]. Subsequently, a phylogenetic tree was constructed with the Minimum Evolution algorithm by using MEGA6 [59]. To investigate gene duplication and loss events, reconciliation analysis was conducted by using Notung software (version 2.6) [60] with the species and gene trees. A total of 34 genomes were subjected to the reconciliation analysis, comprising 25 fungi, one Oomycete, one bacterium, two archaea, two plants, and three animals. Non-fungal species were also included to better understand the evolutionary history (Table 2).

### Multiple sequence alignment, phylogenetic tree construction, and visualization of conserved sequences

Full-length protein sequences of the 442 predicted Dicers were aligned using ClustalW [46]. A phylogenetic tree was constructed using MEGA6 [59] by the Minimum Evolution method with 10,000 bootstrap replicates.

In order to detect conservation of amino acid residues, 84 RdRP sequences were aligned using M-Coffee [61]. One putative RdRP-encoding gene, FOXG_00217 in *Fusarium oxysporum*, was excluded from the analysis because of its very short domain region (68 aa). Sequence logos were created by using WebLogo [62].

## Availability of supporting data

All data described in this paper can be freely accessed at the funRNA website http://funrna.riceblast.snu.ac.kr/ using the latest versions of Google Chrome, Mozilla Firefox, Microsoft Internet Explorer (9 or higher), and Apple Safari. The data sets supporting the results of this article are included in the article and its additional files.

## Competing interests

The authors declare that they have no competing interests.

## Authors' contributions

JC and YHL designed the project. JC developed the pipeline and database. JC and HS developed the web interfaces. JC, KTK, and JJ conducted the data analysis. JC, KTK, JJ, JW, HS, FOA, and YHL wrote the manuscript. All the authors read and approved the final manuscript.

## Supplementary Material

Additional file 1 **Summary of the number of predicted genes encoding Argonaute, Dicer, and RdRP in 1,440 proteomes**. List of 1,440 taxonomically ordered species whose proteomes were scanned with domain profiles, showing the numbers of putative genes for each gene family. Click here for file

Additional file 2 **Partial alignments of catalytic residues (DDH motifs) of Argonautes from 34 species**. Since the DDH motif is interspersed in the Piwi domain, partial alignments near to each catalytic residue are shown. For each cell, the DDH motif is located at the 8th, 14th, and 27th positions (including gaps). Click here for file

Additional file 3 **Phylogenetic tree constructed with 442 Dicer sequences predicted from 180 proteomes**. A total of 442 full-length Dicer sequences were used to construct a phylogenetic tree. The tree could be divided into four major clades, two of which were predominant for animals, and one each for plants and fungi. A Metazoa-dominant clade with minimal domains is shown in pink; the other metazoan clade is shown in red. The Viridiplantae-dominant clade is shown in green and the fungal clade in blue. Click here for file

Additional file 4 **Distribution of auxiliary domains found in 442 Dicer sequences**. Presence and absence table of additional domains found in Dicer sequences. "O" indicates the presence of the corresponding domain and "X" absence. Click here for file

Additional file 5 **Sequence logos based on multiple sequence alignment of 84 putative RdRP sequences**. Sequence logos for double-psi β-barrels (DPBB1 and 2) and the flap sub-domain based on a multiple sequence alignment of 84 sequences including an RdRP from *N. crassa *(QDE-1). Amino acid residues with 70% or more conservation are highlighted in red. Click here for file

## References

[B1] Fire A Xu S Montgomery MK Kostas SA Driver SE Mello CC Potent and specific genetic interference by double-stranded RNA in *Caenorhabditis elegans* Nature 1998 391 6669 806 811 10.1038/35888 9486653

[B2] Carmell MA Xuan ZY Zhang MQ Hannon GJ The Argonaute family: tentacles that reach into RNAi, developmental control, stem cell maintenance, and tumorigenesis Genes Dev 2002 16 21 2733 2742 10.1101/gad.1026102 12414724

[B3] Ding SW Voinnet O Antiviral immunity directed by small RNAs Cell 2007 130 3 413 426 10.1016/j.cell.2007.07.039 17693253 PMC2703654

[B4] Ghildiyal M Zamore PD Small silencing RNAs: an expanding universe Nat Rev Genet 2009 10 2 94 108 10.1038/nrg2504 19148191 PMC2724769

[B5] Li LD Chang SS Liu Y RNA interference pathways in filamentous fungi Cell Mol Life Sci 2010 67 22 3849 3863 10.1007/s00018-010-0471-y 20680389 PMC4605205

[B6] Romano N Macino G Quelling: transient inactivation of gene expression in *Neurospora crassa *by transformation with homologous sequences Mol Microbiol 1992 6 22 3343 3353 10.1111/j.1365-2958.1992.tb02202.x 1484489

[B7] Cogoni C Irelan JT Schumacher M Schmidhauser TJ Selker EU Macino G Transgene silencing of the *al-1 *gene in vegetative cells of *Neurospora *is mediated by a cytoplasmic effector and does not depend on DNA-DNA interactions or DNA methylation EMBO J 1996 15 12 3153 3163 8670816 PMC450258

[B8] Carattoli A Cogoni C Morelli G Macino G Molecular characterization of upstream regulatory sequences controlling the photoinduced expression of the *albino-3 *gene of *Neurospora crassa* Mol Microbiol 1994 13 5 787 795 10.1111/j.1365-2958.1994.tb00471.x 7815938

[B9] Cogoni C Macino G Isolation of quelling-defective (*qde*) mutants impaired in posttranscriptional transgene-induced gene silencing in *Neurospora crassa* Proc Natl Acad Sci USA 1997 94 19 10233 10238 10.1073/pnas.94.19.10233 9294193 PMC23345

[B10] Cogoni C Macino G Gene silencing in *Neurospora crassa *requires a protein homologous to RNA-dependent RNA polymerase Nature 1999 399 6732 166 169 10.1038/20215 10335848

[B11] Cogoni C Macino G Posttranscriptional gene silencing in *Neurospora *by a RecQ DNA helicase Science 1999 286 5448 2342 2344 10.1126/science.286.5448.2342 10600745

[B12] Catalanotto C Pallotta M ReFalo P Sachs MS Vayssie L Macino G Cogoni C Redundancy of the two Dicer genes in transgene-induced posttranscriptional gene silencing in *Neurospora crassa* Mol Cell Biol 2004 24 6 2536 2545 10.1128/MCB.24.6.2536-2545.2004 14993290 PMC355837

[B13] Lee DW Pratt RJ McLaughlin M Aramayo R An argonaute-like protein is required for meiotic silencing Genetics 2003 164 2 821 828 12807800 10.1093/genetics/164.2.821 PMC1462569

[B14] Shiu PK Raju NB Zickler D Metzenberg RL Meiotic silencing by unpaired DNA Cell 2001 107 7 905 916 10.1016/S0092-8674(01)00609-2 11779466

[B15] Volpe TA Kidner C Hall IM Teng G Grewal SI Martienssen RA Regulation of heterochromatic silencing and histone H3 lysine-9 methylation by RNAi Science 2002 297 5588 1833 1837 10.1126/science.1074973 12193640

[B16] Nakayashiki H Kadotani N Mayama S Evolution and diversification of RNA silencing proteins in fungi J Mol Evol 2006 63 1 127 135 10.1007/s00239-005-0257-2 16786437

[B17] Song JJ Smith SK Hannon GJ Joshua-Tor L Crystal structure of Argonaute and its implications for RISC slicer activity Science 2004 305 5689 1434 1437 10.1126/science.1102514 15284453

[B18] Apweiler R Bateman A Martin MJ O'Donovan C Magrane M Alam-Faruque Y Alpi E Antunes R Arganiska J Casanova EB Bely B Bingley M Bonilla C Britto R Bursteinas B Chan WM Chavali G Cibrian-Uhalte E Da Silva A De Giorgi M Fazzini F Gane P Castro LG Garmiri P Hatton-Ellis E Hieta R Huntley R Legge D Liu WD Luo J Activities at the Universal Protein Resource (UniProt) Nucleic Acids Res 2014 42 D1 D191 D198 24253303 10.1093/nar/gkt1140 PMC3965022

[B19] Laurie JD Linning R Bakkeren G Hallmarks of RNA silencing are found in the smut fungus *Ustilago hordei *but not in its close relative *Ustilago maydis* Curr Genet 2008 53 1 49 58 10.1007/s00294-007-0165-7 18060405

[B20] Laurie JD Ali S Linning R Mannhaupt G Wong P Guldener U Munsterkotter M Moore R Kahmann R Bakkeren G Schirawski J Genome comparison of barley and maize smut fungi reveals targeted loss of RNA silencing components and species-specific presence of transposable elements Plant Cell 2012 24 5 1733 1745 10.1105/tpc.112.097261 22623492 PMC3442566

[B21] Drinnenberg IA Weinberg DE Xie KT Mower JP Wolfe KH Fink GR Bartel DP RNAi in Budding Yeast Science 2009 326 5952 544 550 10.1126/science.1176945 19745116 PMC3786161

[B22] Baum J Papenfuss AT Mair GR Janse CJ Vlachou D Waters AP Cowman AF Crabb BS de Koning-Ward TF Molecular genetics and comparative genomics reveal RNAi is not functional in malaria parasites Nucleic Acids Res 2009 37 11 3788 3798 10.1093/nar/gkp239 19380379 PMC2699523

[B23] Mueller AK Hammerschmidt-Kamper C Kaiser A RNAi in *Plasmodium* Curr Pharm Des 2014 20 2 278 283 10.2174/13816128113199990027 23701540

[B24] McRobert L McConkey GA RNA interference (RNAi) inhibits growth of *Plasmodium falciparum* Mol Biochem Parasitol 2002 119 2 273 278 10.1016/S0166-6851(01)00429-7 11814579

[B25] Malhotra P Dasaradhi PVN Kumar A Mohmmed A Agrawal N Bhatnagar RK Chauhan VS Double-stranded RNA-mediated gene silencing of cysteine proteases (falcipain-1 and-2) of *Plasmodium falciparum* Mol Microbiol 2002 45 5 1245 1254 10.1046/j.1365-2958.2002.03105.x 12207693

[B26] Schwentke A Krepstakies M Mueller AK Hammerschmidt-Kamper C Motaal BA Bernhard T Hauber J Kaiser A In vitro and in vivo silencing of plasmodial dhs and eIf-5a genes in a putative, non-canonical RNAi-related pathway BMC Microbiol 2012 12 107 10.1186/1471-2180-12-107 22694849 PMC3438091

[B27] Dalmay T Hamilton A Rudd S Angell S Baulcombe DC An RNA-Dependent RNA polymerase gene in *Arabidopsis *is required for posttranscriptional gene silencing mediated by a transgene but not by a virus Cell 2000 101 5 543 553 10.1016/S0092-8674(00)80864-8 10850496

[B28] Stein P Svoboda P Anger M Schultz RM RNAi: Mammalian oocytes do it without RNA-dependent RNA polymerase RNA-Publ RNA Soc 2003 9 2 187 192 10.1261/rna.2860603 PMC1370384 12554861

[B29] Schroda M RNA silencing in *Chlamydomonas*: mechanisms and tools Curr Genet 2006 49 2 69 84 10.1007/s00294-005-0042-1 16308700

[B30] Zorin B Hegemann P Sizova I Nuclear-gene targeting by using single-stranded DNA avoids illegitimate DNA integration in *Chlamydomonas reinhardtii* Eukaryot Cell 2005 4 7 1264 1272 10.1128/EC.4.7.1264-1272.2005 16002652 PMC1168964

[B31] Wang JC Wang YM Luo D *LjCYC *genes constitute floral dorsoventral asymmetry in *Lotus japonicus* J Integr Plant Biol 2010 52 11 959 970 10.1111/j.1744-7909.2010.00926.x 20977654

[B32] Kumagai H Kinoshita E Ridge RW Kouchi H RNAi knock-down of *ENOD40s *leads to significant suppression of nodule formation in *Lotus japonicus* Plant Cell Physiol 2006 47 8 1102 1111 10.1093/pcp/pcj081 16816411

[B33] Kapoor M Arora R Lama T Nijhawan A Khurana JP Tyagi AK Kapoor S Genome-wide identification, organization and phylogenetic analysis of Dicer-like, Argonaute and RNA-dependent RNA Polymerase gene families and their expression analysis during reproductive development and stress in rice BMC Genomics 2008 9 451 10.1186/1471-2164-9-451 18826656 PMC2576257

[B34] Liu JD Carmell MA Rivas FV Marsden CG Thomson JM Song JJ Hammond SM Joshua-Tor L Hannon GJ Argonaute2 is the catalytic engine of mammalian RNAi Science 2004 305 5689 1437 1441 10.1126/science.1102513 15284456

[B35] Rivas FV Tolia NH Song JJ Aragon JP Liu JD Hannon GJ Joshua-Tor L Purified Argonaute2 and an siRNA form recombinant human RISC Nat Struct Mol Biol 2005 12 4 340 349 10.1038/nsmb918 15800637

[B36] Joshua-Tor L The Argonautes Cold Spring Harb Symp Quant Biol 2006 71 67 72 10.1101/sqb.2006.71.048 17381282

[B37] Meister G Landthaler M Patkaniowska A Dorsett Y Teng G Tuschl T Human Argonaute2 mediates RNA cleavage targeted by miRNAs and siRNAs Mol Cell 2004 15 2 185 197 10.1016/j.molcel.2004.07.007 15260970

[B38] Margis R Fusaro AF Smith NA Curtin SJ Watson JM Finnegan EJ Waterhouse PM The evolution and diversification of Dicers in plants FEBS Lett 2006 580 10 2442 2450 10.1016/j.febslet.2006.03.072 16638569

[B39] Song JJ Liu JD Tolia NH Schneiderman J Smith SK Martienssen RA Hannon GJ Joshua-Tor L The crystal structure of the Argonaute2 PAZ domain reveals an RNA binding motif in RNAi effector complexes Nat Struct Biol 2003 10 12 1026 1032 10.1038/nsb1016 14625589

[B40] Yan KS Yan S Farooq A Han A Zeng L Zhou MM Structure and conserved RNA binding of the PAZ domain Nature 2003 426 6965 469 474 10.1038/nature02129 14615802

[B41] Lingel A Simon B Izaurralde E Sattler M Structure and nucleic-acid binding of the *Drosophila *Argonaute 2 PAZ domain Nature 2003 426 6965 465 469 10.1038/nature02123 14615801

[B42] Tahbaz N Kolb FA Zhang H Jaronczyk K Filipowicz W Hobman TC Characterization of the interactions between mammalian PAZ PIWI domain proteins and Dicer EMBO Rep 2004 5 2 189 194 10.1038/sj.embor.7400070 14749716 PMC1298981

[B43] Salgado PS Koivunen MRL Makeyev EV Bamford DH Stuart DI Grimes JM The structure of an RNAi polymerase links RNA silencing and transcription PLoS Biol 2006 4 12 2274 2281 10.1371/journal.pbio.0040434 PMC1750930 17147473

[B44] Choi J Cheong K Jung K Jeon J Lee GW Kang S Kim S Lee YW Lee YH CFGP 2.0: a versatile web-based platform for supporting comparative and evolutionary genomics of fungi and Oomycetes Nucleic Acids Res 2013 41 D1 D714 D719 10.1093/nar/gks1163 23193288 PMC3531191

[B45] Johnson M Zaretskaya I Raytselis Y Merezhuk Y McGinnis S Madden TL NCBI BLAST: a better web interface Nucleic Acids Res 2008 36 W1 W5 W9 18440982 10.1093/nar/gkn201 PMC2447716

[B46] Larkin MA Blackshields G Brown NP Chenna R McGettigan PA McWilliam H Valentin F Wallace IM Wilm A Lopez R Thompson JD Gibson TJ Higgins DG Clustal W and Clustal X version 2.0 Bioinformatics 2007 23 21 2947 2948 10.1093/bioinformatics/btm404 17846036

[B47] Choi J Park J Kim D Jung K Kang S Lee YH Fungal Secretome Database: Integrated platform for annotation of fungal secretomes BMC Genomics 2010 11 105 10.1186/1471-2164-11-105 20146824 PMC2836287

[B48] Choi J Kim KT Jeon J Lee YH Fungal plant cell wall-degrading enzyme database: a platform for comparative and evolutionary genomics in fungi and Oomycetes BMC Genomics 2013 14 Suppl 5 S7 10.1186/1471-2164-14-S5-S7 24564786 PMC3852112

[B49] Park J Lee S Choi J Ahn K Park B Park J Kang S Lee YH Fungal cytochrome P450 database BMC Genomics 2008 9 402 10.1186/1471-2164-9-402 18755027 PMC2542383

[B50] Cheong K Choi J Choi J Park J Jang S Lee YH Eukaryotic DNAJ/K Database: A Comprehensive Phylogenomic Analysis Platform for the DNAJ/K Family Genomics Inform 2013 11 1 52 54 10.5808/GI.2013.11.1.52 23613683 PMC3630386

[B51] Bailey TL Elkan C Fitting a mixture model by expectation maximization to discover motifs in biopolymers Proc Int Conf Intell Syst Mol Biol 1994 2 28 36 7584402

[B52] Hunter S Jones P Mitchell A Apweiler R Attwood TK Bateman A Bernard T Binns D Bork P Burge S de Castro E Coggill P Corbett M Das U Daugherty L Duquenne L Finn RD Fraser M Gough J Haft D Hulo N Kahn D Kelly E Letunic I Lonsdale D Lopez R Madera M Maslen J McAnulla C McDowall J InterPro in 2011: new developments in the family and domain prediction database Nucleic Acids Res 2012 40 D1 D306 D312 10.1093/nar/gkr948 22096229 PMC3245097

[B53] Roy K Chanfreau GF Guillaume FC, Fuyuhiko T Chapter Ten - The Diverse Functions of Fungal RNase III Enzymes in RNA Metabolism The Enzymes 2012 31 Academic Press 213 235 10.1016/B978-0-12-404740-2.00010-0 27166447

[B54] Hu Y Stenlid J Elfstrand M Olson A Evolution of RNA interference proteins dicer and argonaute in Basidiomycota Mycologia 2013 105 6 1489 1498 10.3852/13-171 23928424

[B55] Xu Z Hao BL CVTree update: a newly designed phylogenetic study platform using composition vectors and whole genomes Nucleic Acids Res 2009 37 W1 W174 W178 19398429 10.1093/nar/gkp278 PMC2703908

[B56] Wang H Xu Z Gao L Hao BL A fungal phylogeny based on 82 complete genomes using the composition vector method BMC Evol Biol 2009 9 195 10.1186/1471-2148-9-195 19664262 PMC3087519

[B57] Zuo G Xu Z Yu H Hao B Jackknife and bootstrap tests of the composition vector trees Genomics Proteomics Bioinformatics 2010 8 4 262 267 10.1016/S1672-0229(10)60028-9 21382595 PMC5054193

[B58] Felsenstein J PHYLIP (Phylogeny Inference Package) version 3.6 Distributed by the author 2005 Department of Genome Sciences, University of Washington, Seattle

[B59] Tamura K Stecher G Peterson D Filipski A Kumar S MEGA6: Molecular Evolutionary Genetics Analysis Version 6.0 Mol Biol Evol 2013 30 12 2725 2729 10.1093/molbev/mst197 24132122 PMC3840312

[B60] Chen K Durand D Farach-Colton M NOTUNG: A program for dating gene duplications and optimizing gene family trees J Comput Biol 2000 7 3-4 429 447 10.1089/106652700750050871 11108472

[B61] Wallace IM O'Sullivan O Higgins DG Notredame C M-Coffee: combining multiple sequence alignment methods with T-Coffee Nucleic Acids Res 2006 34 6 1692 1699 10.1093/nar/gkl091 16556910 PMC1410914

[B62] Crooks GE Hon G Chandonia JM Brenner SE WebLogo: A sequence logo generator Genome Res 2004 14 6 1188 1190 10.1101/gr.849004 15173120 PMC419797

