# Supplementary figures and images for: funRNA: a fungi-centered genomics platform for genes encoding key components of RNAi
Source: BMC Genomics. 2014 Dec 8;15(Suppl 9):S14. doi: 10.1186/1471-2164-15-S9-S14 (PMC4290597; doi:10.1186/1471-2164-15-S9-S14)

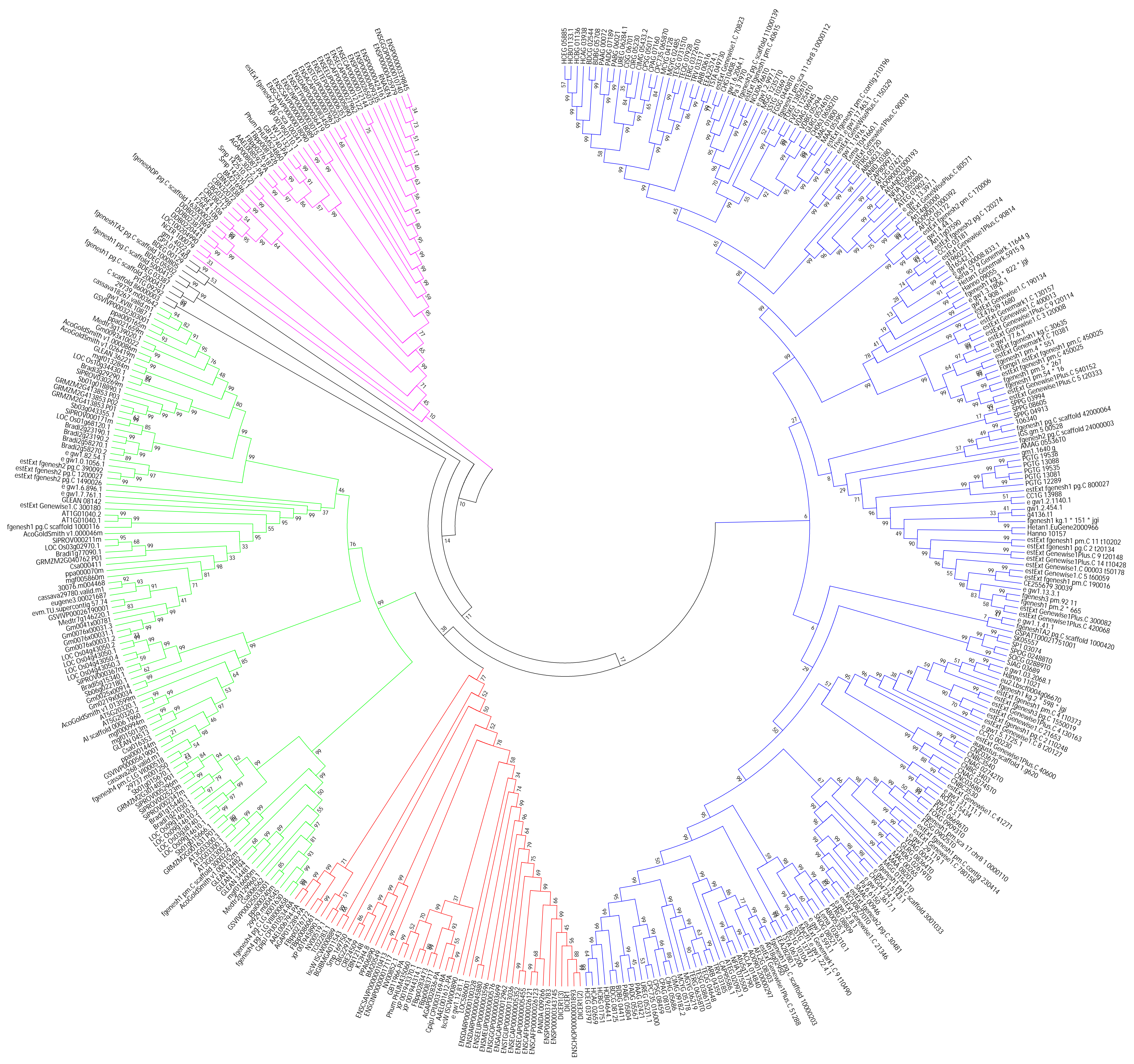

Supplement: Additional file 3 — Phylogenetic tree constructed with 442 Dicer sequences predicted from 180 proteomes. A total of 442 full-length Dicer sequences were used to construct a phylogenetic tree. The tree could be divided into four major clades, two of which were predominant for animals, and one each for plants and fungi. A Metazoa-dominant clade with minimal domains is shown in pink; the other metazoan clade is shown in red. The Viridiplantae-dominant clade is shown in green and the fungal clade in blue. [file 1471-2164-15-S9-S14-S3.PDF]

# DPBB1 of QDE-1

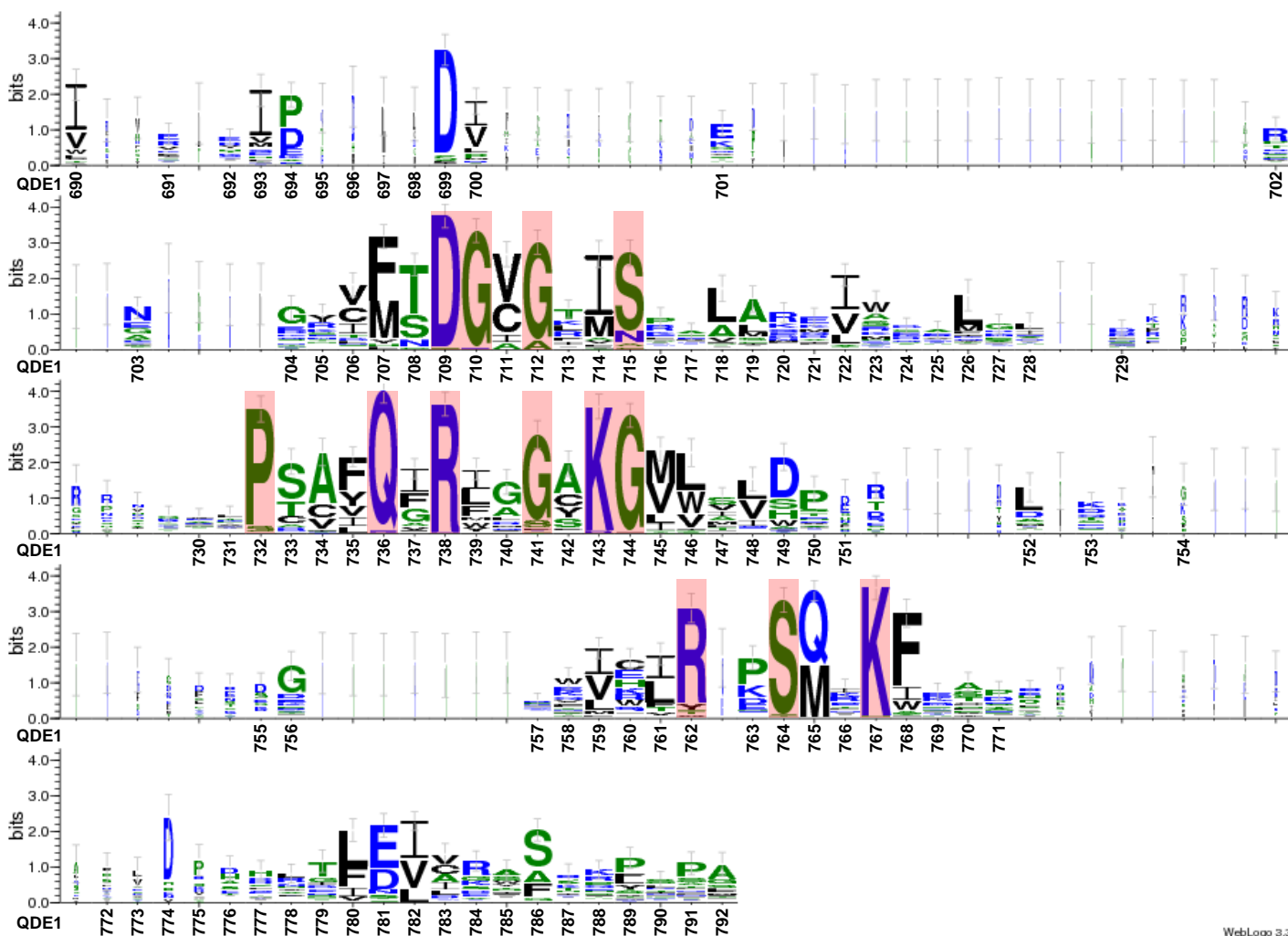

# DPBB2 of QDE-1

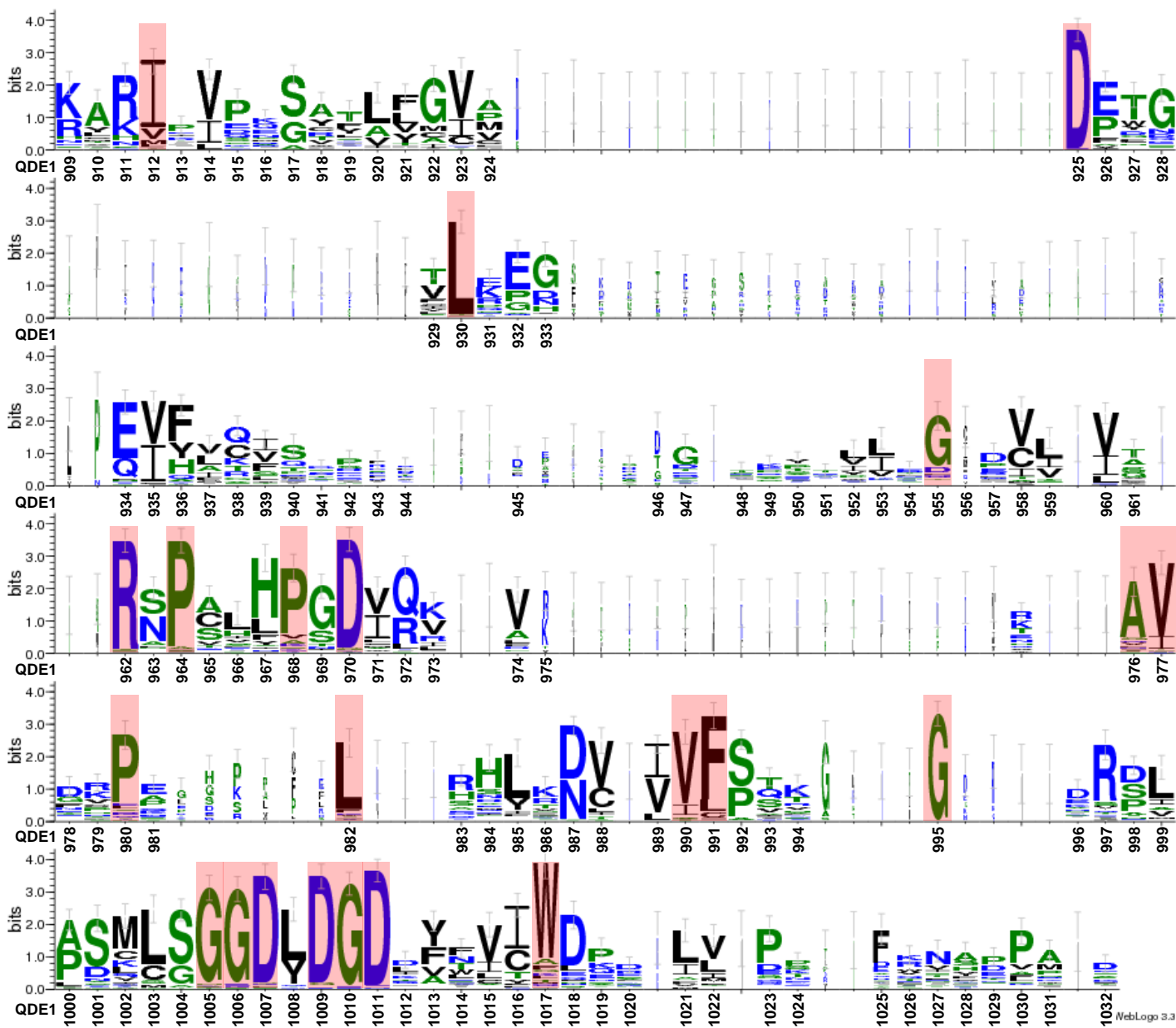

# Flap sub-domain of QDE-1

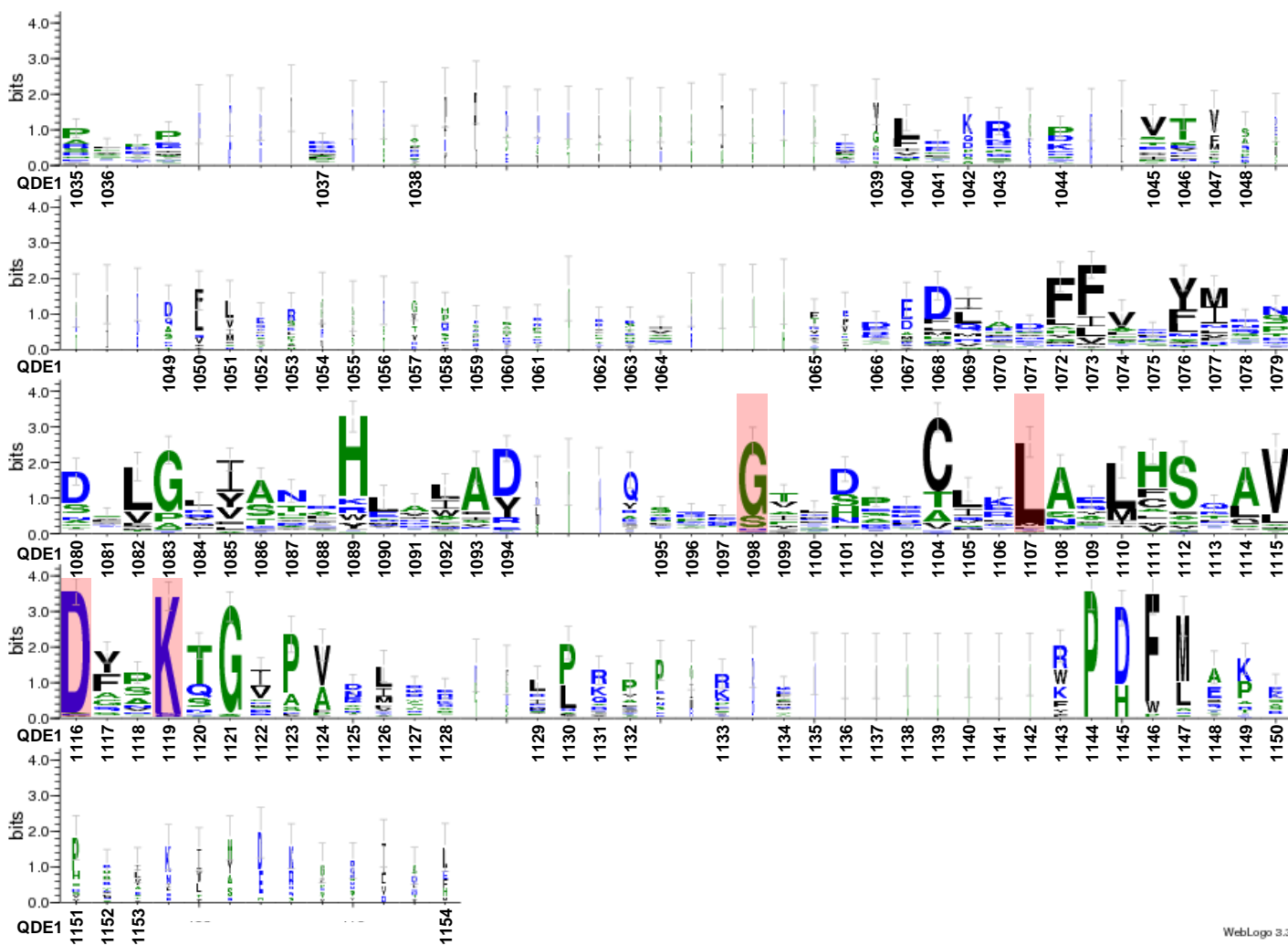

Supplement: Additional file 5 — Sequence logos based on multiple sequence alignment of 84 putative RdRP sequences. Sequence logos for double-psi β-barrels (DPBB1 and 2) and the flap sub-domain based on a multiple sequence alignment of 84 sequences including an RdRP from N. crassa (QDE-1). Amino acid residues with 70% or more conservation are highlighted in red. [file 1471-2164-15-S9-S14-S5.PDF]
